# The smallest in the deepest: the enigmatic role of viruses in the deep biosphere

**DOI:** 10.1093/nsr/nwad009

**Published:** 2023-01-10

**Authors:** Lanlan Cai, Markus G Weinbauer, Le Xie, Rui Zhang

**Affiliations:** State Key Laboratory of Marine Environmental Science, College of Ocean and Earth Sciences, Xiamen University, Xiamen 361102, China; Department of Ocean Science, The Hong Kong University of Science and Technology, Hong Kong, China; Sorbonne Universités, UPMC, Université Paris 06, CNRS, Laboratoire d’Océanographie de Villefranche (LOV), Villefranche BP28, France; State Key Laboratory of Marine Environmental Science, College of Ocean and Earth Sciences, Xiamen University, Xiamen 361102, China; State Key Laboratory of Marine Environmental Science, College of Ocean and Earth Sciences, Xiamen University, Xiamen 361102, China; Institute for Advanced Study, Shenzhen University, Shenzhen 518055, China

**Keywords:** virus, deep biosphere, population size, diversity, activity, ecological role

## Abstract

It is commonly recognized that viruses control the composition, metabolism, and evolutionary trajectories of prokaryotic communities, with resulting vital feedback on ecosystem functioning and nutrient cycling in a wide range of ecosystems. Although the deep biosphere has been estimated to be the largest reservoir for viruses and their prokaryotic hosts, the biology and ecology of viruses therein remain poorly understood. The deep virosphere is an enigmatic field of study in which many critical questions are still to be answered. Is the deep virosphere simply a repository for deeply preserved, non-functioning virus particles? Or are deep viruses infectious agents that can readily infect suitable hosts and subsequently shape microbial populations and nutrient cycling? Can the cellular content released by viral lysis, and even the organic structures of virions themselves, serve as the source of bioavailable nutrients for microbial activity in the deep biosphere as in other ecosystems? In this review, we synthesize our current knowledge of viruses in the deep biosphere and seek to identify topics with the potential for substantial discoveries in the future.

## Teaser

Unexpected highly abundant, active, and diverse viruses have been observed in marine and terrestrial subsurface environments. The potential ecological and biogeochemical roles of viruses in the deep biosphere are discussed in this review paper.

The deep biosphere, which is the largest ecosystem on Earth, generally refers to the collection of habitats that persist at least one meter below the surface of continents and the bottom of oceans [1,2]. Sediments and igneous basements with active fluid flux are major geological settings for the marine subseafloor biome, whereas sedimentary rocks and aquifer systems are the main habitats for life in the terrestrial deep biosphere [3]. A consensus is that these physically and chemically varied habitats are dominated by a large number and diversity of prokaryotes and their viruses [4]. Given that the deep biosphere harbors more than half of the Earth's microbes and is the largest reservoir of carbon and nitrogen, microbial processes in the deep biosphere are believed to strongly affect the biogeochemical cycles of our planet [5].

Viral infection is a potential major biological driver influencing ecological processes and biogeochemical cycles mediated by microbes [6,7]. As the most abundant and diverse component in all ecosystems investigated on Earth, the critical roles of viruses in microbial abundance, diversity, activity, and ecosystem functioning have been well recognized in surface systems such as oceans, lakes, soil, and shallow sediments [8–11]. Viruses can influence microbial communities and nutrient availability through top-down control by lysing host cells and bottom-up control by releasing labile cellular contents that fuel the growth of non-infected cells [8,12,13]. Viruses also mediate the diversity and evolution of their hosts through a multitude of processes such as horizontal gene transfer, selection for resistance, and reprogramming of metabolism [14,15]. Although the story therein is still emerging, evidence is beginning to show that viruses are also abundant in the deep biosphere, with the numbers always higher than those of their microbial counterparts [16–18]. According to the most recent census, the deep biosphere hosts an estimated >7 × 10^30^ viruses, rivalling the estimated totals in aquatic systems, shallow sediments, and soil [19,20]. However, whether—and if so, how—the massive viral effects on the dynamics of microbial populations and nutrient cycling universally apply to the deep biosphere remains an open question.

Considering the huge size of the deep biosphere and the key role that viruses play in regulating microbes on a global scale, it is critically important to understand the reasons for viral prevalence and diversity, know the real scenario of viral infection and their interaction with microbial hosts, and figure out the implications for ecology and biogeochemistry in the deep biosphere. In this review, we summarize the current knowledge of the deep virosphere in both terrestrial and oceanic regimes, discuss the potential ecological and biogeochemical effects of viruses in the deep biosphere based on the richer knowledge from aquatic ecosystems, and identify some topics with the potential for substantial discoveries and progress in future research.

## DEEP BIOSPHERE: THE LARGEST RESERVOIR FOR VIRUSES

**Figure 1.**
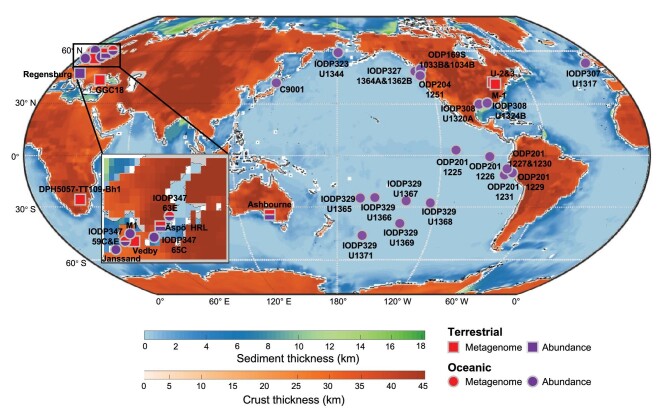
Study sites of viruses in the deep biosphere. Dots and rectangles denote the studies in oceanic and terrestrial regions, respectively. Purple indicates the sampling area where the quantification of virus population size is available. Red indicates the sampling area where investigations of viruses have been carried out using genomic data. The inset map shows the Baltic Sea Basin.

**Figure 2.**
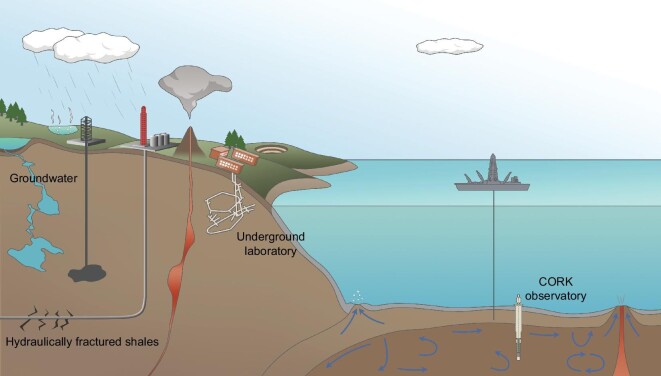
Typical deep environments where viral studies have been performed so far.

In recent years, the understanding of the deep virosphere has expanded because of more opportunities of sampling for microbiology in deep environments and the gradual recognition of the vital impact of viruses on microbes in various ecosystems. Studies related to viruses in the marine deep biosphere have relied mainly on the Ocean Drilling Program (ODP) and Integrated Ocean Drilling Program, which have contributed to most of the knowledge about viruses in the marine deep biosphere. In the terrestrial deep biosphere, although a variety of natural settings are present, studies of viruses are often limited to groundwater [21–26] and fracture fluid systems [27–29] (Figs 1 and 2).

**Figure 3.**
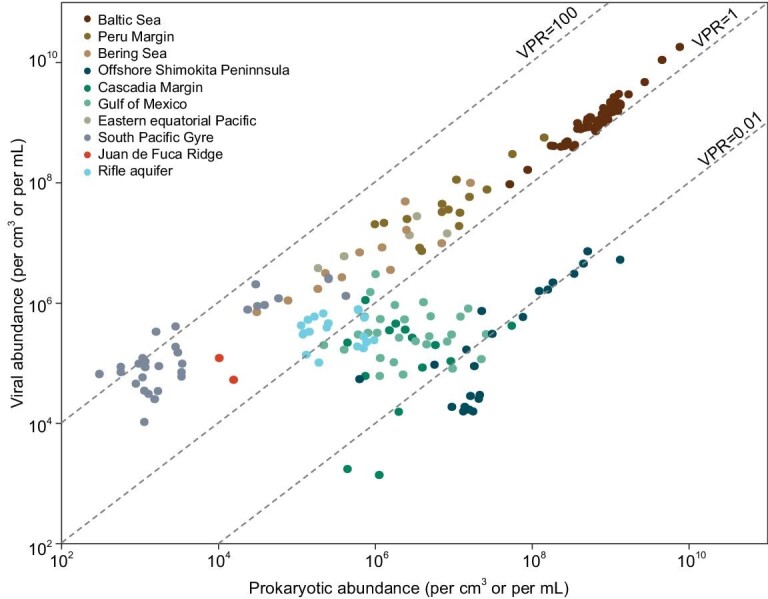
Relationship between viruses and prokaryotic host cells in the deep biosphere. VPR, virus-to-prokaryote ratio.

The earliest report of viruses in the subseafloor was by Bird *et al*. [16], who investigated viral abundance throughout the entire Holocene to late-Pleistocene sediments in the Saanich Inlet, Canada (ODP Leg 169S). Viral abundance reached 10^9^ g^−1^ in the anoxic sediments down to 118 meters below seafloor (mbsf) (corresponding to a sediment age of *c*.14 kiloyears (ky)) [16]. Similarly, virus densities up to 10^10^ cm^−3^ occurred in the young, organic-rich sediments of the Baltic Sea Basin and North Sea tidal-flat (corresponding to a sediment age of thousands of years) (Fig. 3) [18,30]. Based on ODP Leg 201, the first ocean drilling expedition dedicated to the study of life beneath the seafloor, viral numbers as high as 10^6^–10^8^ cm^−3^ were recorded in the sediments from the continental margin of Peru and the equatorial Pacific Ocean (down to 381 mbsf, *c*.15 million years ago (Ma)) [30]. Similar values were found in the sediments of the Porcupine Seabight area in the North Atlantic Ocean and the northeastern Bering Sea slope [17,30]. By contrast, older and more oligotrophic subseafloor sediments harbor orders of magnitude fewer viruses. For example, the abyssal oligotrophic sediments of the South Pacific Gyre have viral abundances of 10^3^–10^7^ cm^−3^ [30]. So far, the oldest and deepest samples with virus particles that have been quantified are the Eocene sediments from the low-productivity South Pacific Gyre, which is >50 Ma, and the Pleistocene sediments from the Gulf of Mexico down to a depth of more than 600 mbsf [30,31]. Virus particles have also been detected in the oceanic basement fluids from the Juan de Fuca Ridge in the Northeast Pacific, with abundances of 10^4^–10^5^ mL^−1^ [32]. The upper part of the fluids sampling sites is covered with basement rock up to 240 m thick, which prevents the exchange of fluids with the overlying seawater, implying that viruses in the fluids are likely to be indigenous communities and the voluminous oceanic crust is a habitable, but almost unexplored, environment for viruses [32].

Compared with the amounts of information available for virus studies within the subseafloor, much less information is available for the terrestrial deep virosphere, with virus abundance measurements having been performed in only a handful of aquifer systems. From 10^5^–10^7^ mL^−1^ virus-like particles were found in 69–450 m deep igneous rock aquifers that contained recent Baltic Sea–influenced groundwater to ancient glacial meltwaters with ages from months to thousands of years old [21]. Similarly, virus abundances of 10^4^–10^6^ mL^−1^ have been observed in hydrologically and geologically distinct aquifer systems, such as the uranium-impacted alluvial aquifer of the Rifle River, USA, and the 15–90-m deep aquifer waters at Ashbourne, South Australia [24–26].

Taken together, these studies show that viruses are widely distributed in the deep biosphere and are usually present in high numbers (Fig. 3). Summed globally, the total number of viruses in the deep subseafloor is estimated to be 5 × 10^30^, compared with approximately 2 × 10^30^ viruses in the terrestrial deep biosphere [19]. The oceanic crust, which is much less explored [32], was estimated to harbor 0.2–2 × 10^30^ viruses with a high degree of uncertainty [19]. Considering the number of viruses found in the oceanic basement, it is expected that the scope of the deep virosphere will extend vertically with the advances in sampling techniques. Furthermore, a re-counting study based on an improved method for separating viruses from subseafloor sediments showed two times and even up to 350 times more viruses being extracted than the originally published results, indicating the population size of viruses in the deep biosphere could be larger [33]. These findings suggest that the deep biosphere may be the largest reservoir for viruses on Earth, compared to the globally estimated total numbers of 1.5–2 × 10^30^ and 6.2 × 10^29^ for marine environment [19,20] and soil [19], respectively. Such a high abundance of viruses may affect microbial ecological characteristics and processes, which could influence biogeochemical cycling within the deep biosphere and on a global scale.

The factors affecting the population size and biogeographic distribution of viruses varied across habitats due to the high heterogeneity of deep environments. Given the parasitism of viruses, the number of viruses typically varies with the abundance and productivity of their hosts (i.e. prokaryotes in the deep biosphere). Consequently, in the deep biosphere, the population size of viruses generally decreased with increasing depth and age, following the trend of prokaryotic abundance. The virus-to-prokaryote ratio (VPR), which is an index that reflects the balance between the population sizes of viruses and their hosts, indicates the general virus–host interaction at the population level. In the deep biosphere, VPRs of 225 and 0.001 are the highest and lowest that have been reported so far (Fig. 3). For marine sediments, despite the total abundances of viruses and prokaryotes declining by several orders of magnitude with increasing depth, a constant VPR, albeit with varying values across locations, generally occurred in the first tens of meters below the seafloor. For example, only small variations in VPRs (1.1–3.5) were observed throughout the organic-rich sediments (down to tens of meters) in the Baltic Sea and Saanich Inlet, where the rapid sedimentation resulted in thick young sediments (thousands of years) [16,18]. Relatively stable VPRs (1–10 with no significant difference) were also recorded within the first 100 m of sediments from the continental margin of Peru, the northeastern Bering Sea slope, and the Porcupine Seabight area [17,30]. These constant VPRs indicate a stable balance between the production and removal of viruses and their hosts in the upper and young sediments. Higher VPRs were observed in the deeper, older, and more oligotrophic sediments with fewer viruses and cells. VPRs of 10–23 were reported in sediments from the slope sites of Peru and the Bering Sea at depths of 100–320 m [17,30]. Remarkably, the abyssal oligotrophic sediments of the South Pacific Gyre, which have low sediment rates, had much higher VPRs of up to 225 detected at 36 mbsf [30]. This result suggested that viral particles were better preserved than prokaryotic cells in the deep and ancient sediments, maybe due to the higher adsorption of virions onto the sediment matrix and decreased degradation by exoenzymes [34].

## DIVERSITY OF DEEP VIRUSES: AN UNEXPLORED GENETIC REPOSITORY

**Figure 4.**
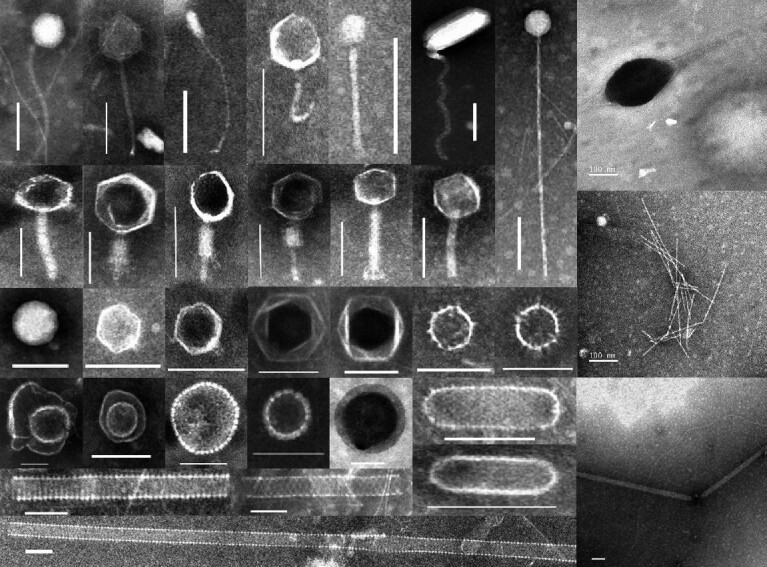
Transmission electron microscopy micrographs showing morphologies of virus-like particles observed in the deep Baltic Sea biosphere. Scale bar: 100 nm [18].

In the deep biosphere, not only do viruses occur in large numbers, but they also show remarkable diversity in morphology and genetic content. Transmission electron microscopy studies of viral-like particles have discovered a wealth of morphotypes and provided hints about their host communities (Fig. 4). Along with the explicit identification of tailed morphology commonly associated with bacterial viruses (*Siphoviridae, Myoviridae, Podoviridae*), a variety of virus-like particles with typical archaeal virion morphotypes, such as spherical, spindle-shaped (lemon-shaped), and rod-shaped, have been observed in the deep habitats where archaea could constitute a substantial proportion of the microbial community [18,21,32]. The untailed icosahedral particles with different diameters could be viruses that infect hosts in different domains [18,21]. The larger ones (diameters >100 nm) are morphologically characteristic of members of the family *Phycodnaviridae*, which infect eukaryotic algae, and the family *Iridoviridae*, which infect a diverse array of invertebrate hosts, whereas the smaller ones could be bacterial viruses such as those from family *Tectiviridae*. It is unsurprising to find viruses that probably originated from the photosynthetic zone, which may be vertically transported to and preserved (and colonized if habitable) in the deep biosphere [18,28,35]. Furthermore, it is likely that, besides the potential exogenous input, the diverse viral forms are also indicative of special viral communities thriving in the deep biosphere.

The study of viral genetic diversity and the evolutionary implications started almost one decade after the discovery of viruses in the deep biosphere [35,36]. Unlike in other environments (e.g. surface aquatic and soil ecosystems), viral genetic information in the deep biosphere has rarely been studied by viromics (targeted metagenomics of virus-like particles) by which presumptive virions are separated from cellular organisms before nucleic acid extraction. This may be largely due to the difficulty in obtaining sufficient samples and the challenge of recovering enough viral DNA/RNA without using biased amplification approaches. The few currently available viromic datasets are from the deep groundwaters of three Äspö Hard Rock Laboratory wells [23] and three Hainich Critical Zone Exploratory aquifer wells [37]. Other studies generally mined viral sequences from metagenomic data generated from microbial assemblages (including both viruses and cells) using bioinformatic tools, which had the limitation of detecting only the highly abundant virus signals [27,28,32,38]. These approaches have detected a vast diversity of viruses and provided insights into virus–host interactions (e.g. the infection strategy of viruses and co-evolution of hosts and viruses, see discussion below) in the deep biosphere [27,28,32,38]. Usually, large numbers of viral contigs that cannot be matched to any known viruses are obtained. Most of the known deep viral sequences are tailed double-stranded DNA viruses (families *Myoviridae, Podoviridae, Siphoviridae*; order Caudovirales) that infect mainly bacteria and archaea (e.g. [27,28]). Sequences from double-stranded DNA viruses that infect algae (e.g. *Phycodnaviridae*), amoeba (e.g. *Marseilleviridae* and *Mimiviridae*), and even invertebrates (e.g. *Iridoviridae* and *Poxviridae*) have also been detected (e.g. [37]). Single-stranded DNA viruses (e.g. *Microviridae* and *Circoviridae*) seem to be a minority group in deep groundwater viromes [37], but they may constitute a significant proportion of viral communities in subseafloor sediments as revealed by metatranscriptomic study [39]. An analysis of virus-like sequences in metagenomes from oceanic fluids, which were covered by 3.5-million-year-old basaltic crust, also found a distinct assemblage of viruses, most of which potentially infect archaea [32]. This result implies that the sediment-covered basalt harbored a plethora of viral lineages that were different from those of known viruses. The pronounced diversity of Altiarchaeota viruses, for which most of the genes were unannotated, has also been reported in four terrestrial groundwater ecosystems across three continents [38]. Together, these findings show a highly unique viral community in the deep biosphere that is unlike those in other environments or among known viral isolates. Another interesting finding was the dissimilarity of viral communities in different deep environments, which indicates that the selection of unique virus populations adapted to different deep biosphere environments takes place [27]. For example, 156 previously undescribed candidate viral genera (accounting for 46% of the predicted viral population) were obtained from a metagenomics study of five deep terrestrial subsurface locations (hydraulically fractured shales), and only 17 of these genera were shared across all five locations, suggesting the geographical separation of viral assemblages in the terrestrial deep biosphere [27].

So far, only a few isolated viruses have been characterized due to the limited number of hosts from the deep biosphere and the strict requirements for cultivation in most cases. Sequencing of isolated viruses and induced proviruses has enriched the genetic catalog of viruses in the deep biosphere. The sequences of two dozen viruses and induced proviruses, which infect typical bacteria lineages from deep environments, including bacteria in genera *Halanaerobium, Pseudodesulfovibrio, Pseudomonas*, and *Rhizobium*, have been obtained (Table 1). In the genome sequences of two temperate phages of *R. radiobacter* P007 from deep subseafloor sediments, >70% of the predicted genes were unknown [40]. Similarly, the recovery of two novel bacteriophage genera from groundwater highlights the potential of terrestrial subsurface environments as huge reservoirs of underexplored viruses [41].

**Table 1.** Isolated phages and induced proviruses from the deep biosphere.

Habitat	Depth (mbsf)	Host	Phage name	Classification	Life strategy	Genome size (kb)	Reference
Groundwater	7–12	*Bacillus mycoides*	Anath	*Siphoviridae*	Lytic	52	[41]
Groundwater	7–12	*Pseudomonas* sp.	Lana	*Siphoviridae*	Lytic	88	[41]
Groundwater	183–455	*Pseudodesulfovibrio aespoeensis*	HEy 1–5	*Podoviridae*	Lytic	NA	[22]
Sediment	6.1–19.5	*Pseudomonas* sp. Alda10	NA	Filamentous	Lysogenic	NA	[44]
Hydraulic fracturing shale	2500	*Halanaerobium congolense* WG8	NA	Non-tailed	Lysogenic	45	[27]
Sediment	198	*Rhizobium radiobacter* P007	NA	*Myoviridae*	Lysogenic	30	[36]
Sediment	320	*Vibrio diazotrophicus* R6	NA	*Myoviridae*	Lysogenic	100; 125	[36]
Sediment	1	*Vibrio diazotrophicus* P082	NA	*Myoviridae*	Lysogenic	55; 75	[36]
Sediment	268	*Rhodobacter capsulatus* E32	NA	*Siphoviridae*	Lysogenic	35	[36]
Sediment	43	*Rhodovulum sulfidophilum* P122A	NA	*Siphoviridae*	Lysogenic	36	[36]
Sediment	198	*Paenibacillus glucanolyticus* P073A	NA	*Siphoviridae*	Lysogenic	38–95	[36]
Sediment	198	*Rhizobium radiobacter* P007	RR1-A	NA	Lysogenic	52	[40]
Sediment	198	*Rhizobium radiobacter* P007	RR1-B	NA	Lysogenic	37	[40]

mbsf, meters below surface for terrestrial deep biosphere, meters below seafloor for marine deep biosphere; NA, not available.

Currently, it is difficult to estimate the genetic richness of viruses in the deep biosphere due to sporadic sampling. The results could be influenced by the types of samples (i.e. fluids or solids) and the approaches used (e.g. metagenome, single cell, and cultivation). Despite this limitation, it is almost certain that the deep virosphere contains unique but as yet unknown viral communities in which different environmental stresses have resulted in site-specific genetic diversity. The detection of novel viruses, including those that are less abundant, is becoming more feasible with the combination of deeper sequencing and more isolation efforts, as well as improved and more sophisticated bioinformatic methods. Furthermore, the dynamics of viral community composition and the effects of ecological processes on/during viral community changes are yet to be studied in detail to understand how virus communities are generated and evolve in the deep biosphere.

## ACTIVITY OF DEEP VIRUSES: LIVING OR DORMANT

Viruses can be extracellular as free virus particles (termed virions) or intracellular within infected host cells. As virions, which is the form that is usually quantified in the environment, virus particles can be viewed as a chemical complex of mainly nucleic acids and proteins. In marine ecosystems, it is estimated that 10^28^ viral infections occur daily [8]. Generally, viral infection increases with increasing host cell density because infection is a direct function of virus–host encounters. Considering that most of the environments in the deep biosphere (e.g. in sediment pores) typically have co-localized viruses and prokaryotes with specific concentrations [17,18,30,31], the probability of physical contact between a virus and a host cell could be high [42], implying that most deep biosphere habitats are potentially favorable environments for viral infection. However, the issue of whether viruses in the deep biosphere are active or not remains controversial and still poorly studied owing to methodological challenges.

Some data have suggested that viral infection may be common in the deep biosphere. RNA transcripts from both subseafloor and terrestrial groundwaters showed the expression of many viral homologues, indicating viral activity within the deep biosphere [23,27,28,38,39]. An analysis of the composition of a CRISPR-Cas (clustered regularly interspaced short palindromic repeats-CRISPR associated protein) array showed the continued development of CRISPR spacers (1–8 spacers) in the metagenome-assembled genomes of *Halanaerobium* during the sampling period of only 216 days, indicating frequent and continuous viral infection in deep terrestrial fracturing systems [27]. The CRISPR analysis also showed that more than half of the viruses might infect multiple *Halanaerobium* hosts, suggesting a broad host range [27]. Recently, virus fluorescence *in situ* hybridization (VirusFISH), which uses virus-targeted gene probes and allows the visualization of intracellular virus infection [43], demonstrated the lytic infection of archaeal viruses in a dense biofilm from a sulfidic spring in Bavaria, Germany [38]. In addition, Cai and colleagues observed visibly infected cells by electron microscopy in samples from deep Baltic Sea sediments down to 70 mbsf (*c*. thousands of years old) [18]. Despite these findings, so far, only one study has quantified the activity of viruses in the deep biosphere, and one more has quantified the activity of viruses in terrestrial subsurface environments [18,44]. Lytic viral infection of 10^5^–10^7^ cm^−3^ h^−1^ was recorded in the deep Baltic Sea sediments down to 37 mbsf by dilution-based incubation [18]. The high virus infection measured in the Baltic Sea sediments may be the result of organic-rich and microbially dense conditions and/or the presumably stimulated viral lysis during incubation.

It is also inevitable that viruses will lose activity during their transportation and persistence in deep environments. Adsorption of virions to mineral and organic particles may destroy or mask the receptors for viral attachment onto host cell surfaces and cause the inactivation of the viruses. However, the attachment with particles also helps viruses survive longer because of the protective effects against inactivation by enzyme digestion [45]. In addition, it has been shown that the inactivation of viruses in groundwater can be reversible under certain conditions when chemical components stabilize the virus particles and the capsid proteins may rebound to the host cells [46]. Therefore, abiotic factors related to viral adsorption such as temperature, pH, salinity, sediment/soil type, organic matter concentration, and water content are considered to affect viral activity and decay (e.g. [45]). Furthermore, the activity and stability of viral particles may be influenced by biotic factors; for example, the activity of heterotrophic prokaryotes and extracellular enzymes [47]. Proteases and nucleases released by microorganisms may degrade the viral capsid and nucleic acid, and such processes are influenced by the morphological characteristics (e.g. capsid size) of viruses and environmental factors such as temperature [48]. So far, no direct measurement of the inactivation or decay rate of viral communities in the deep biosphere has been reported. If the viral population size is stable, as was shown in the upper deep-sea sediment [34], viral decay should be balanced by viral production, and the viral decay rate will be similar to the viral production rate.

## INFECTION STRATEGY: TO KILL OR NOT TO KILL

**Figure 5.**
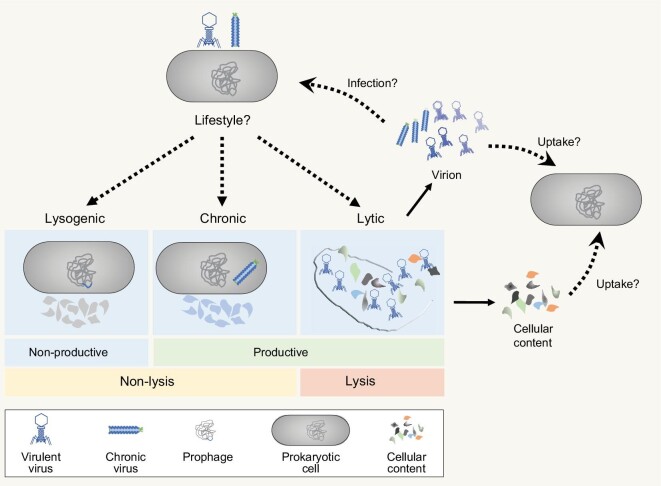
Viral processes and key questions to be addressed in the deep biosphere. After successful entry into the host cell, virus can have a productive replication cycle that results in the release of new virions with cell lysis (lytic infection) or without cell lysis (chronic infection). Alternatively, virus can enter a non-productive cycle (lysogenic infection), in which virus integrates its genome into the host chromosome. Key issues that remain to be resolved in the deep biosphere (shown by dashed lines) include: (1) the frequency of viral infection; (2) the assessment of virus lifestyles; (3) the bioavailability of the cellular content released by viral lysis and virions.

After successful entry into a host cell, viruses replicate mainly by lytic, chronic, or lysogenic infection processes (Fig. 5). In lytic or chronic infection, virus redirects the host metabolism toward the production and release of progeny viruses, leading to lysis (lytic infection) or non-lysis (chronic infection) of the host cell. In lysogenic infection, the genome of temperate virus (referred to as prophage) is integrated into the host cell chromosome or is maintained as a plasmid until an induction event triggers either the lytic or chronic cycle [49]. The type of viral replication cycle depends on multiple factors but is supposed to depend mostly on the environmental conditions and host metabolic status. Lytic infection is generally thought to be more common in locations and times of high host density and productivity, whereas lysogenic infection is prevalent in environments where host abundance is too low and/or where hosts are inactive for viral populations to be maintained by lytic infection [50], such as deep sedimentary environments.

Prokaryotic genomic studies of the deep biosphere frequently detect prophage-like sequences in the isolate genomes and metagenome-assembled sequences (e.g. [51]). Several active prophages have been found in the deep biosphere by induction assays of microbial isolates [27,36,40,44] (Table 1). Treatment with mitomycin C, an inducing agent, indicated the presence of prophages in 46% of the bacterial isolates in the marine deep subseafloor sediments from the Eastern Equatorial Pacific and the Peru Margin [36]. Similarly, all isolates of the subseafloor species *R. radiobacter*, which accounted for an average of 0.75% of the total bacterial population, were lysogens [40]. Mitomycin C also induced filamentous viral-like particles without cell lysis from the nitrate-reducing bacterium *Pseudomonas* sp. Alda10 from alluvial aquifer sediment [44]. Daly *et al*. successfully induced active prophages of *Halanaerobium* in deep engineered terrestrial fracturing systems using the organic acid succinate and the heavy metal copper chloride [27]. Additionally, viral sequences identified as putative prophages made up 24.9% of the viral pool in the same terrestrial deep environment based on a metagenomic analysis [27]. Currently, the only study to quantify the lysogenic infection rate in the deep biosphere suggested that lysogenic viral production accounted for <40% of the total viral production in the organic-rich Baltic Sea subseafloor [18]; however, the potential stimulation effects of lysogeny into lysis during the *ex-situ* sample processing could not be excluded. Nevertheless, these studies indicate that lysogenic infection is a major viral proliferation mode in the deep biosphere. There are very few studies about phages associated with chronic infection in the deep biosphere, which are mostly filamentous phages belonging to the family *Inoviridae*. Homologues of filamentous phage sequences have been identified in metatranscriptomic data of deep subseafloor sediments from the Peruvian margin [39], indicating chronic infection without lysis of host cells may be present in deep subseafloor sediments.

Undoubtedly, more studies are needed, including the further development of methods and more measurements in different habitats, for a quantitative assessment of virus lifestyles (i.e. the relative frequency of lysogeny and lysis) to understand host–virus ecological interactions in the deep biosphere. Beyond this, determining the relationship between virus lifestyle and environmental physicochemical conditions is needed to obtain a clearer picture of how viruses mediate microbial mortality and affect the global nutrient cycle in deep environments.

## HOST–VIRUS INTERACTIONS: FRIEND OR FOE

On the cellular level, viruses can trigger a response in their hosts, resulting in complex and dynamic host–virus interactions, and such interactions could also happen in the deep biosphere. Viruses were considered parasites that rely entirely on their hosts and were regarded as the natural enemies of microbes. However, it is currently recognized that viruses are not necessarily virulent and that viral infection can sometimes be ‘beneficial’ for their hosts in certain infection strategies [49]. Lytic viruses are commonly regarded as killers, forming an antagonistic interaction with their hosts. The evolutionary arms race between viruses and their hosts, which results in ever-evolving anti-viral strategies and viral responses, is the often-invoked example of an antagonistic relationship. This relationship was demonstrated in samples from deep terrestrial hydraulically fractured wells by a time series analysis of CRISPR spacers in hosts and protospacers in viruses at the lineage level [27]. In this long-term monitoring study, the crash of *Halanaerobium* populations in hydraulically fractured shales was found to be closely coupled with the decrease in the relative abundance of *Halanaerobium*-associated viruses, which are probably the only ‘predators’ of *Halanaerobium* in this ecosystem [27]. Additionally, one *Halanaerobium* metagenome-assembled genome showed a total of 43 spacers with 20 links to the same viral sequence, which indicated the critical roles that active viral infection played in driving microbial community development [27].

Generally, lysogenic and chronic viruses have been considered to be more mutualistic with their hosts. Several studies have indicated that prophages contribute to the survival of hosts in stressed environments by inducing beneficial changes in the physiology of the host and/or the suppression of unnecessary metabolic activities [52,53]. Furthermore, temperate viruses render their hosts immune to secondary infection through a process called superinfection exclusion [54]. An excellent example of virus–host mutualistic interaction was discovered recently, where chronically virus-infected archaeon *Sulfolobus islandicus* cells killed the virus-resistant cells in the population by producing a protein toxin that was resistant by virus-infected cells [55]. This process removed competitor cells in the population for the host and also ensured the persistence of the virus in the context of highly distributed immunity [55]. From the virus perspective, superinfection exclusion minimizes within-host virus–virus competition and ensures the continued vertical transmission of viruses. Being intracellular can also provide a survival advantage for viruses under unfavorable conditions such as low-susceptibility host density, low host activity, and high temperature, which are commonly observed in the deep biosphere. Understanding host–virus interactions in relevant scenarios from the deep biosphere will help in understanding the implications of the ecological role of viruses in the ecosystem of deep environments.

## ROLE OF VIRUSES IN THE DEEP ECOSYSTEM: TOP-DOWN OR BOTTOM-UP

The limited observational data that are currently available provide a glimpse into the potential ecological role of viruses in the deep ecosystem. The deep biosphere is a highly compact and mostly anoxic environment that generally lacks multicellular grazers for prokaryotes, implying that viral lysis may be the main cause of mortality that controls microbial population size and turnover in deep subsurface sediments, thereby playing a more important top-down role than it does in surface ecosystems and deep sea [11]. Viral predation could be beneficial to the ecosystem by avoiding the overpopulation of certain host species and maintaining genetic diversity, which can help ecosystems to be more resilient [49,54]. Even though viral lysis leads to the death of specific hosts, it is thought to stimulate the growth of the remaining community members through the regeneration of nutrients and the release of ecological niches, such as the temporal community shift on the *Halanaerobium* genotype level observed in the hydraulic fracturing ecosystem [27]. Thus, lytic viruses can act as top-down controllers, killing the winner and possibly driving the diversity of the host community. Quantification of deep viral infection in Baltic Sea deep sediment resulted in the potential turnover times of prokaryotes induced by viral lysis of 2.3–16.2 days [18]. These values are comparable to those obtained from deep-sea surface sediments (<2–67 days) but much shorter than cell turnover times measured by other methods (e.g. amino acid racemization and cell-specific metabolic rate), which ranged from tens to hundreds of years [6]. If active viral infections frequently occurred in a large volume of the deep virosphere, the ecological consequence would be immense through their collective activities. Conversely, if viruses fail to infect hosts because of, for example, adsorption, which may be a common process for viruses in the deep biosphere, then the role of viruses in controlling microbial communities extrapolated from surface systems might be limited in the deep biosphere.

In addition, viral lysates could support the growth of non-infected bacteria and archaea, thereby acting as a bottom-up controller for host community assembly [56]. Danczak and coworkers [29] found that bacterial and viral community assemblies within fractured shale ecosystems were coordinated by different ecological processes, possibly because of a dynamic feedback loop between the top-down and bottom-up effects of viruses. The new progeny virions, as well as empty capsids that remain outside the host cell after infection, are also potential sources of nutrients and energy for heterotrophs (Fig. 5). Viral particles have been shown to undergo fast decomposition and contribute to significant amounts of carbon, nitrogen, and phosphate pools in deep-sea sediments where active extracellular enzymes exist [34]. Although there are no data on the decay products of viruses and their bioavailability, viral particles may be decomposed and available to be used for the growth of heterotrophic microorganisms, serving as bottom-up factors in the deep ecosystem.

The redistribution and partitioning of inorganic and organic materials by viral lysis of host cells have been termed the ‘viral shunt’ in the ocean [57]. Viruses have been estimated to shunt about 145 gigatons of carbon, 27.6 gigatons of nitrogen, and 4.6 gigatons of phosphate annually in deep tropical and subtropical oceans [58]. The quantitative assessment of the viral role in the cycling of carbon and other elements in the deep biosphere is currently precluded by a paucity of data. Cai *et al*. [18] calculated that the carbon released by potential viral lysis in the Baltic Sea deep sediments ranged from 0.03–0.81 μg cm^−3^ d^−1^ (0.01–0.30 × 10^−18^ gigatons per cm^3^ annually). Although this is a relatively small and possibly overestimated amount compared with the total amount of organic matter, the viral lysates generally showed higher bioavailability than the recalcitrant organic matter that is not easily utilized by microbes after long-term sedimentation. Bradley *et al*. estimated that the oxidation of a single dead cell per year provides sufficient power to support the maintenance demands of dozens to thousands of cells in marine sediments, especially in relatively young sediments (<10 000 years) [59]. Therefore, virus-recycled organic carbon may support the maintenance demands of non-infected cells in the low-energy deep biosphere [18].

Besides the effect of lysis on biogeochemical cycling, viruses may reprogram the metabolism of host cells during lytic or lysogenic infection (i.e. virocell, the ‘living form’ of the virus within infected host cells) [60,61]. Diverse virus-encoded auxiliary metabolic genes (vAMGs) involved in biogeochemical cycling were discovered in different environments, including deep-sea and surface sediments [62,63]. For the deep biosphere, vAMGs of sulfur cycling, including sulfate assimilation (e.g. phosphoadenosine phosphosulfate and adenylyl-sulfate kinase) and thiosulfate oxidation (e.g. sulfatase), have been observed in the deep groundwaters of three Äspö Hard Rock Laboratory wells [23]. Considering that vAMGs involved in carbon (e.g. carbohydrate metabolism) [64], nitrogen (e.g. nitrification *amoC* genes) [65], and phosphate (e.g. *phoH*) [66] processes are frequently detected in the deep sea, hydrothermal vent, sediment, and soil samples, it is reasonable to suppose that such vAMGs are present in viruses in the deep biosphere and serve as key agents in modulating microbial activities and biogeochemical processes.

## CONCLUDING REMARKS AND FUTURE DIRECTIONS

In the past two decades, an understanding has developed that many viruses are present in the deep biosphere and that the deep biosphere cannot be properly understood without considering such viruses. However, only the tip of the iceberg in the deep virosphere has been touched till now. Many key issues remain to be resolved (Fig. 5).

1. Difficulties in sampling and methodology have limited studies to a few sites, thereby biasing the current understanding. More research efforts in dissecting the distribution and diversity of viruses in different habitats are needed to gain a more comprehensive view of the scale and content of the deep virosphere. This important study direction can be achieved with advances in sampling techniques and more sampling opportunities and will likely deliver many correlative datasets.
2. Another major challenge is that knowledge about the activity of viruses and how viruses interact with their hosts is still patchy and conjectural. Taking advantage of the advanced cultivation approaches with recently developed molecular techniques (e.g. metagenomics, single-cell sequencing) and *in-situ* visualization (e.g. VirusFISH) may provide essential insights into the function of viruses in the deep biosphere.
3. Furthermore, understanding the extent to which viral infection alters microbial community composition and organic matter pools, incorporating virus-mediated processes into biogeochemical models of the deep biosphere, and extrapolating the consequences for nutrient fluxes to a global scale will contribute to a more complete conceptual model of life and biogeochemical turnover from micro to ecosystem scales.

## References

[1] Edwards KJ , Becker K, Colwell F. The deep, dark energy biosphere: intraterrestrial life on Earth. Annu Rev Earth Planet Sci 2012; 40: 551–68. 10.1146/annurev-earth-042711-105500

[2] Hoehler TM , Jørgensen BB. Microbial life under extreme energy limitation. Nat Rev Microbiol 2013; 11: 83–94. 10.1038/nrmicro2939 23321532

[3] Colwell FS , D’Hondt S. Nature and extent of the deep biosphere. Rev Mineral Geochem 2013; 75: 547–74. 10.2138/rmg.2013.75.17

[4] Jørgensen BB , Boetius A. Feast and famine—microbial life in the deep-sea bed. Nat Rev Microbiol 2007; 5: 770–81. 10.1038/nrmicro1745 17828281

[5] D’Hondt S , Pockalny R, Fulfer VM et al. Subseafloor life and its biogeochemical impacts. Nat Commun 2019; 10: 1–13. 10.1038/s41467-019-11450-z 31388058 PMC6684631

[6] Jørgensen BB , Andren T, Marshall IPG. Sub-seafloor biogeochemical processes and microbial life in the Baltic Sea. Environ Microbiol 2020; 22: 1688–706. 10.1111/1462-2920.14920 31970880

[7] Anderson RE , Brazelton WJ, Baross JA. The deep viriosphere: assessing the viral impact on microbial community dynamics in the deep subsurface. Rev Mineral Geochem 2013; 75: 649–75. 10.2138/rmg.2013.75.20

[8] Suttle CA . Marine viruses—major players in the global ecosystem. Nat Rev Microbiol 2007; 5: 801–12. 10.1038/nrmicro1750 17853907

[9] Danovaro R , Dell’Anno A, Corinaldesi C et al. Major viral impact on the functioning of benthic deep-sea ecosystems. Nature 2008; 454: 1084–7. 10.1038/nature07268 18756250

[10] Lopez-Bueno A , Tamames J, Velazquez D et al. High diversity of the viral community from an Antarctic lake. Science 2009; 326: 858–61. 10.1126/science.1179287 19892985

[11] Zhang R , Li Y, Yan W et al. Viral control of biomass and diversity of bacterioplankton in the deep sea. Commun Biol 2020; 3: 256. 10.1038/s42003-020-0974-5 32444696 PMC7244761

[12] Suttle CA . Viruses in the sea. Nature 2005; 437: 356–61. 10.1038/nature04160 16163346

[13] Breitbart M , Bonnain C, Malki K et al. Phage puppet masters of the marine microbial realm. Nat Microbiol 2018; 3: 754–66. 10.1038/s41564-018-0166-y 29867096

[14] Xu Y , Zhang R, Wang N et al. Novel phage–host interactions and evolution as revealed by a cyanomyovirus isolated from an estuarine environment. Environ Microbiol 2018; 20: 2974–89. 10.1111/1462-2920.14326 30051557

[15] Thompson LR , Zeng Q, Kelly L et al. Phage auxiliary metabolic genes and the redirection of cyanobacterial host carbon metabolism. Proc Natl Acad Sci USA 2011; 108: E757–64. 10.1073/pnas.1102164108 21844365 PMC3182688

[16] Bird DF , Juniper SK, Ricciardi-Rigault M et al. Subsurface viruses and bacteria in Holocene/Late Pleistocene sediments of Saanich Inlet, BC: ODP Holes 1033B and 1034B, Leg 169S. Mar Geol 2001; 174: 227–39. 10.1016/S0025-3227(00)00152-3

[17] Middelboe M , Glud RN, Filippini M. Viral abundance and activity in the deep sub-seafloor biosphere. Aquat Microb Ecol 2011; 63: 1–8. 10.3354/ame01485

[18] Cai L , Jørgensen BB, Suttle CA et al. Active and diverse viruses persist in the deep sub-seafloor sediments over thousands of years. ISME J 2019; 13: 1857–64. 10.1038/s41396-019-0397-9 30877284 PMC6776017

[19] Bar-On YM , Phillips R, Milo R. The biomass distribution on Earth. Proc Natl Acad Sci USA 2018; 115: 6506–11. 10.1073/pnas.1711842115 29784790 PMC6016768

[20] Xie L , Wei W, Cai L et al. A global viral oceanography database (gVOD). Earth Syst Sci Data 2021; 13: 1251–71. 10.5194/essd-13-1251-2021

[21] Kyle JE , Eydal HSC, Ferris FG et al. Viruses in granitic groundwater from 69 to 450 m depth of the Äspö hard rock laboratory, Sweden. ISME J 2008; 2: 571–4. 10.1038/ismej.2008.18 18288217

[22] Eydal HSC , Jägevall S, Hermansson M et al. Bacteriophage lytic to *Desulfovibrio aespoeensis* isolated from deep groundwater. ISME J 2009; 3: 1139–47. 10.1038/ismej.2009.66 19516280

[23] Holmfeldt K , Nilsson E, Simone D et al. The Fennoscandian Shield deep terrestrial virosphere suggests slow motion ‘boom and burst’ cycles. Commun Biol 2021; 4: 307. 10.1038/s42003-021-01810-1 33686191 PMC7940616

[24] Pan D , Nolan J, Williams KH et al. Abundance and distribution of microbial cells and viruses in an alluvial aquifer. Front Microbiol 2017; 8: 1199. 10.3389/fmicb.2017.01199 28744257 PMC5504356

[25] Roudnew B , Lavery TJ, Seymour JR et al. Spatially varying complexity of bacterial and virus-like particle communities within an aquifer system. Aquat Microb Ecol 2013; 68: 259–66. 10.3354/ame01615

[26] Roudnew B , Seymour JR, Jeffries TC et al. Bacterial and virus-like particle abundances in purged and unpurged groundwater depth profiles. Groundwater Monit R 2012; 32: 72–7. 10.1111/j.1745-6592.2011.01393.x

[27] Daly RA , Roux S, Borton MA et al. Viruses control dominant bacteria colonizing the terrestrial deep biosphere after hydraulic fracturing. Nat Microbiol 2019; 4: 352–61. 10.1038/s41564-018-0312-6 30510171

[28] Daly RA , Borton MA, Wilkins MJ et al. Microbial metabolisms in a 2.5-km-deep ecosystem created by hydraulic fracturing in shales. Nat Microbiol 2016; 1: 16146. 10.1038/nmicrobiol.2016.146 27595198

[29] Danczak RE , Daly RA, Borton MA et al. Ecological assembly processes are coordinated between bacterial and viral communities in fractured shale ecosystems. mSystems 2020; 5: e00098–20. 10.1128/mSystems.00098-20 32184367 PMC7380583

[30] Engelhardt T , Kallmeyer J, Cypionka H et al. High virus-to-cell ratios indicate ongoing production of viruses in deep subsurface sediments. ISME J 2014; 8: 1503–9. 10.1038/ismej.2013.245 24430483 PMC4069387

[31] Yanagawa K , Morono Y, Yoshida-Takashima Y et al. Variability of subseafloor viral abundance at the geographically and geologically distinct continental margins. FEMS Microbiol Ecol 2014; 88: 60–8. 10.1111/1574-6941.12269 24308555

[32] Nigro OD , Jungbluth SP, Lin HT et al. Viruses in the oceanic basement. mBio 2017; 8: e02129–16. 10.1128/mBio.02129-16 28270584 PMC5340873

[33] Pan D , Morono Y, Inagaki F et al. An improved method for extracting viruses from sediment: detection of far more viruses in the subseafloor than previously reported. Front Microbiol 2019; 10: 878. 10.3389/fmicb.2019.00878 31110497 PMC6501758

[34] Dell’Anno A , Corinaldesi C, Danovaro R. Virus decomposition provides an important contribution to benthic deep-sea ecosystem functioning. Proc Natl Acad Sci USA 2015; 112: E2014–9. 10.1073/pnas.1422234112 25848024 PMC4413343

[35] Coolen MJL . 7000 Years of *Emiliania huxleyi* viruses in the Black Sea. Science 2011; 333: 451–2. 10.1126/science.1200072 21778399

[36] Engelhardt T , Sahlberg M, Cypionka H et al. Induction of prophages from deep-subseafloor bacteria. Environ Microbiol Rep 2011; 3: 459–65. 10.1111/j.1758-2229.2010.00232.x 23761308

[37] Kallies R , Hölzer M, Brizola Toscan R et al. Evaluation of sequencing library preparation protocols for viral metagenomic analysis from pristine aquifer groundwaters. Viruses 2019; 11: 484. 10.3390/v11060484 31141902 PMC6631259

[38] Rahlff J , Turzynski V, Esser SP et al. Lytic archaeal viruses infect abundant primary producers in Earth's crust. Nat Commun 2021; 12: 4642. 10.1038/s41467-021-24803-4 34330907 PMC8324899

[39] Engelhardt T , Orsi WD, Jørgensen BB. Viral activities and life cycles in deep subseafloor sediments. Environ Microbiol Rep 2015; 7: 868–73. 10.1111/1758-2229.12316 26109514

[40] Engelhardt T , Sahlberg M, Cypionka H et al. Biogeography of *Rhizobium radiobacter* and distribution of associated temperate phages in deep subseafloor sediments. ISME J 2013; 7: 199–209. 10.1038/ismej.2012.92 22855213 PMC3526171

[41] Hylling O , Carstens AB, Kot W et al. Two novel bacteriophage genera from a groundwater reservoir highlight subsurface environments as underexplored biotopes in bacteriophage ecology. Sci Rep 2020; 10: 11879. 10.1038/s41598-020-68389-1 32681144 PMC7368026

[42] Weinbauer MG , Bettarel Y, Cattaneo R et al. Viral ecology of organic and inorganic particles in aquatic systems: avenues for further research. Aquat Microb Ecol 2009; 57: 321–41. 10.3354/ame01363 27478304 PMC4962909

[43] Castillo YM , Sebastian M, Forn I et al. Visualization of viral infection dynamics in a unicellular eukaryote and quantification of viral production using virus fluorescence *in situ* hybridization. Front Microbiol 2020; 11: 1559. 10.3389/fmicb.2020.01559 32765451 PMC7379908

[44] Pan D , Watson R, Wang D et al. Correlation between viral production and carbon mineralization under nitrate-reducing conditions in aquifer sediment. ISME J 2014; 8: 1691–703. 10.1038/ismej.2014.38 24671088 PMC4817613

[45] Kimura M , Jia Z-J, Nakayama N et al. Ecology of viruses in soils: past, present and future perspectives. Soil Sci Plant Nutr 2008; 54: 1–32. 10.1111/j.1747-0765.2007.00197.x

[46] Alvarez ME , Aguilar M, Fountain A et al. Inactivation of MS-2 phage and poliovirus in groundwater. Can J Microbiol 2000; 46: 159–65. 10.1139/w99-128 10721484

[47] Mojica KD , Brussaard CP. Factors affecting virus dynamics and microbial host–virus interactions in marine environments. FEMS Microbiol Ecol 2014; 89: 495–515. 10.1111/1574-6941.12343 24754794

[48] Tian Y , Cai L, Xu Y et al. Stability and infectivity of allochthonous viruses in deep sea: a long-term high pressure simulation experiment. Deep Sea Res Part I 2020; 161: 103302. 10.1016/j.dsr.2020.103302

[49] Correa AMS , Howard-Varona C, Coy SR et al. Revisiting the rules of life for viruses of microorganisms. Nat Rev Microbiol 2021; 19: 501–13. 10.1038/s41579-021-00530-x 33762712

[50] Breitbart M . Marine viruses: truth or dare. Annu Rev Mar Sci 2012; 4: 425–48. 10.1146/annurev-marine-120709-142805 22457982

[51] Labonté JM , Field EK, Lau M et al. Single cell genomics indicates horizontal gene transfer and viral infections in a deep subsurface Firmicutes population. Front Microbiol 2015; 6: 349. 10.3389/fmicb.2015.00349 25954269 PMC4406082

[52] Paul JH . Prophages in marine bacteria: dangerous molecular time bombs or the key to survival in the seas? ISME J 2008; 2: 579–89. 10.1038/ismej.2008.35 18521076

[53] Williamson SJ , Cary SC, Williamson KE et al. Lysogenic virus–host interactions predominate at deep-sea diffuse-flow hydrothermal vents. ISME J 2008; 2: 1112–21. 10.1038/ismej.2008.73 18719614

[54] Chevallereau A , Pons BJ, van Houte S et al. Interactions between bacterial and phage communities in natural environments. Nat Rev Microbiol 2022; 20: 49–62. 10.1038/s41579-021-00602-y 34373631

[55] DeWerff SJ , Bautista MA, Pauly M et al. Killer archaea: virus-mediated antagonism to CRISPR-immune populations results in emergent virus-host mutualism. mBio 2020; 11: e00404–20. 10.1128/mBio.00404-20 32345641 PMC7188992

[56] Zhang R , Weinbauer MG, Qian PY. Viruses and flagellates sustain apparent richness and reduce biomass accumulation of bacterioplankton in coastal marine waters. Environ Microbiol 2007; 9: 3008–18. 10.1111/j.1462-2920.2007.01410.x 17991029

[57] Wilhelm SW , Suttle CA. Viruses and nutrient cycles in the sea. Bioscience 1999; 49: 781–8. 10.2307/1313569

[58] Lara E , Vaqué D, Sà EL et al. Unveiling the role and life strategies of viruses from the surface to the dark ocean. Sci Adv 2017; 3: e1602565. 10.1126/sciadv.1602565 28913418 PMC5587022

[59] Bradley JA , Amend JP, LaRowe D E. Necromass as a limited source of energy for microorganisms in marine sediments. J Geophys Res Biogeosci 2018; 123: 577–90. 10.1002/2017JG004186

[60] Rosenwasser S , Ziv C, Creveld SG et al. Virocell metabolism: metabolic innovations during host–virus interactions in the ocean. Trends Microbiol 2016; 24: 821–32. 10.1016/j.tim.2016.06.006 27395772

[61] Forterre P . The virocell concept and environmental microbiology. ISME J 2013; 7: 233–6. 10.1038/ismej.2012.110 23038175 PMC3554396

[62] Jian H , Yi Y, Wang J et al. Diversity and distribution of viruses inhabiting the deepest ocean on Earth. ISME J 2021; 15: 3094–110. 10.1038/s41396-021-00994-y 33972725 PMC8443753

[63] Kieft K , Breister AM, Huss P et al. Virus-associated organosulfur metabolism in human and environmental systems. Cell Rep 2021; 36:109471. 10.1016/j.celrep.2021.109471 34348151

[64] Jin M , Guo X, Zhang R et al. Diversities and potential biogeochemical impacts of mangrove soil viruses. Microbiome 2019; 7: 58. 10.1186/s40168-019-0675-9 30975205 PMC6460857

[65] Ahlgren NA , Fuchsman CA, Rocap G et al. Discovery of several novel, widespread, and ecologically distinct marine *Thaumarchaeota* viruses that encode *amoC* nitrification genes. ISME J 2019; 13: 618–31. 10.1038/s41396-018-0289-4 30315316 PMC6462027

[66] Wang L , Zhao J, Wang Z et al. *phoH*-carrying virus communities responded to multiple factors and their correlation network with prokaryotes in sediments along Bohai Sea, Yellow Sea, and East China Sea in China. Sci Total Environ 2022; 812: 152477. 10.1016/j.scitotenv.2021.152477 34952046

